# Genetic Variations in the *KCNJ5* Gene in Primary Aldosteronism Patients from Xinjiang, China

**DOI:** 10.1371/journal.pone.0054051

**Published:** 2013-01-31

**Authors:** Nan-Fang Li, Hong-Jian Li, De-Lian Zhang, Ju-Hong Zhang, Xiao-Guang Yao, Hong-Mei Wang, Suofeiya Abulikemu, Ke-Ming Zhou, Xiang-Yang Zhang

**Affiliations:** 1 The Center of Hypertension of the People’s Hospital of Xinjiang Uygur Autonomous Region; The Center of Diagnosis, Treatment and Research of Hypertension in Xinjiang, Urumqi, China; 2 Xinjiang Medical University, Urumqi, China; University of Central Florida, United States of America

## Abstract

**Background:**

Primary aldosteronism (PA) is the most common endocrine form of secondary hypertension, and one of the most common subtypes of sporadic PA is aldosterone-producing adenoma (APA). Recently, two somatic mutations of the *KCNJ5* gene were implicated in APA, and two germline mutations were associated with familial hyperaldosteronism III.

**Objectives:**

This case-control study was designed to investigate the relationship between genetic variations in the *KCNJ5* gene and sporadic PA patients in Xinjiang, China.

**Methods:**

Five common single nucleotide polymorphisms (SNPs) of the *KCNJ5* gene (rs6590357, rs4937391, rs3740835, rs2604204, and rs11221497) were detected in patients with sporadic PA (n = 235) and essential hypertension (EH; n = 913) by the *Taq*Man polymerase chain reaction method.

**Results:**

The EH group and the PA group showed significant differences in the distributions of genotypes and alleles of rs4937391 and rs2604204 in total and male subjects (*P*<0.05), as well as rs3740835 in male subjects (*P*<0.05). However, only the association between the rs2604204 genotype and male sporadic PA remained significant after Bonferroni’s correction (*P*<0.01). Furthermore, logistic regression analysis demonstrated that the CC genotype of rs2604204 was a risk factor for male patients with sporadic PA, after adjusting for age and body mass index (odds ratio = 2.228, 95% CI: 1.300–3.819, *P* = 0.004).

**Conclusion:**

The genetic variant rs2604204 of *KCNJ5* is associated with sporadic PA in Chinese males, suggesting that *KCNJ5* may be involved in the pathogenesis of sporadic PA in these particular patients.

## Introduction

Primary aldosteronism (PA) is a potentially curable disorder characterized by the autonomous overproduction of aldosterone from the adrenal zona glomerulosa. It is the most common endocrine form of secondary hypertension, with an estimated prevalence as high as 10% in patients with hypertension [1]. Compared with essential hypertension (EH) patients, PA patients have a higher incidence of cardiovascular, cerebrovascular and kidney damage [2]–[6]. Five subtypes of PA are currently recognized, including the familial forms, known as familial hyperaldosteronism (FH; types I-III), and four sporadic forms, idiopathic hyperplasia (IHA; caused by bilateral adrenal zona glomerulosa hyperplasia), aldosterone-producing adenoma (APA), primary unilateral adrenal hyperplasia, and pure aldosterone-producing adrenocortical carcinoma. While the two sporadic forms are by far the most common, accounting for ∼95% of all diagnosed PA cases, the exact pathogeneses of IHA and APA remain largely unknown. Elucidation of the underlying molecular mechanisms of these disorders will likely aid in the development of targeted treatments and improved prognosis for PA patients.

Aldosterone is known to play a key role in regulating blood pressure and maintaining electrolyte and fluid homeostasis. However, excessive aldosterone secretion can result in hypertension, low plasma renin, and hypokalemia. Aldosterone secretion from the adrenal zona glomerulosa cells is regulated by serum potassium. Genetic deletion of TWIK-related acid sensitive K+ channels (known as TASK1) not only leads to low-renin hypertension, attributed to the autonomous overproduction of aldosterone, but also a marked depolarization of the adrenal zona glomerulosa cell membrane potential [7]–[8]. Thus, potassium channels are believed to play a central role in regulating the membrane potential of glomerulosa cells.

The *KCNJ5* gene encodes a potassium inwardly rectifying channel (subfamily 1, member 5), which is a member of the G protein-sensitive inwardly rectifying potassium (KG) channel family. The subunit composition of the KG channel family members varies among different cells and tissues, corresponding to their diverse functional roles [9]. Kir3.4 is capable of forming a heterotetrameric channel complex with Kir3.1 in atrial cells to generate the acetylcholine-induced inwardly rectifying K^+^ current (IK_ACh_) [10]–[12].

Studies of the underlying genetic factors of atrial fibrillation have identified two *KCNJ5* gene mutations, rs6590357 and rs7118833, associated with this cardiac event [13]–[14]. Further studies of *KCNJ5* gene variants have found that two somatic mutations (G151R and L168R) and two germline mutations (T158A and G151E) are associated with APA and FH-III, respectively [15]–[19]. However, the relationship between *KCNJ5* polymorphisms and sporadic PA are less well studied. In order to better understand the role of *KCNJ5* genomic variation in sporadic PA, five common *KCNJ5* polymorphisms (rs6590357, rs4937391, rs3740835, rs2604204 and rs11221497) were detected in sporadic PA patients from Xinjiang, China.

## Subjects and Methods

### Ethics Approval of the Study Protocol

The study was approved by the Ethics Committee of the People’s Hospital of Xinjiang Uygur Autonomous Region. All procedures were conducted according to the standards of the Declaration of Helsinki. All study participants provided signed informed consent.

### Study Subjects

A total of 1356 consecutive hypertensive patients were recruited from the inpatient population at the Hypertension Center of the People’s Hospital of Xinjiang Uygur Autonomous Region from January 2010 to December 2010. Patients were excluded from the study if they were diagnosed with FH, adrenocortical carcinoma or any other secondary forms of hypertension, or if they had hypertension combined with congestive heart failure or renal function insufficieny. The final study number was 1148, which including 235 PA and 913 EH patients.

PA diagnosis was made upon demonstration of inappropriate autonomous hypersecretion of aldosterone, as determined by a salt loading assay [20]. Briefly, patients presenting with Joint National Commission VII (JNC 7) stage 2, stage 3, or drug-resistant hypertension, either hypertension with unexplained spontaneous or diuretic-induced hypokalemia, adrenal incidentaloma or hyperplasia, were administered the PA screening test. The patients were requested to discontinue diuretics and mineralocorticoid antagonists for at least six weeks, or angiotensin-converting enzyme inhibitors, angiotensin receptor blockers, dihydropyridines calcium antagonist, and β-receptor blockers for at least four weeks prior to hospital admission. When required, the patients were allowed to continue regimens of verapamil (slow-release) and doxazosin mesylate/terazosin hydrochloride to control high blood pressure. After admission, the seated plasma renin activity (PRA) and serum aldosterone were assayed. Patients with low PRA (<1 ng/dL/ng/rnl/h) combined with a high serum aldosterone level (>12 ng/dL) or serum aldosterone concentration (ng/dL)/plasma renin activity (ng/mL/h) ratio (ARR) >20 underwent an intravenous saline load test. If the post-saline aldosterone level was >5 ng/dL, PA was diagnosed.

All PA patients underwent an adrenal computed tomography scan. In addition, 120 PA patients underwent adrenal vein sampling to assess the presence of unilateral or bilateral adrenal disease in the absence of ACTH stimulation. A cortisol-corrected aldosterone ratio from the high side to the low side of more than 2∶1 was considered to indicate unilateral aldosterone excess, as suggested by the Endocrine Society Guidelines [1]. Finally, 116 cases were successfully subtyped (including 77 bilateral cases and 39 unilateral cases); the remaining 119 patients were not subtyped.

Hypertension diagnosis was made according to the criteria published by the JNC 7. In particular, patients were required to have one of the following features: at least three occasions of systolic blood pressure (SBP) ≥140 mmHg and/or diastolic blood pressure (DBP) ≥90 mmHg, or previous history of hypertension and/or current treatment with antihypertensive medication despite blood pressure readings <140/90 mmHg. After excluding other forms of secondary hypertension through systemic examination, all patients who did not meet the criteria for PA were diagnosed as EH.

### Hormonal and Biochemical Assays

Following an overnight fast, blood samples were drawn from an indwelling catheter positioned in an antecubital vein after 30 min of rest in the sitting position between the hours of 8∶00 AM and 10∶00 AM. Serum or plasma from each blood sample were separated within 1 h after blood collection, and all samples were stored at −20°C until use. In addition, isolated blood cells were stored at −80°C until subsequent genotyping.

Serum aldosterone was measured by radioimmunoassay using a commercially available kit (Beckman Coulter, USA), and the intra- and inter-assay coefficients of variation were 4.5% and 9.8%, respectively. PRA was measured by an iodine [^125^I] angiotensin I radioimmunoassay kit (Northern Biotechnology Institutes, China), and the intra- and inter-assay coefficients of variation were 10% and 15%, respectively. Serum cortisol was measured by the iodine [^125^I] cortisol radioimmunoassay kit (Northern Biotechnology Institutes, China), and the intra- and inter-assay coefficients of variation were 10% and 15%, respectively. Serum sodium, potassium, and creatinine levels were measured on a C800 automated biochemistry analyzer (Abbott Laboratories, USA).

### Single Nucleotide Polymorphism (SNP) Selection and Genotyping

Five SNPs (rs6590357, rs4937391, rs3740835, rs2604204 and rs11221497) were selected from the National Center for Biotechnology Information (NCBI) dbSNP and the International HapMap Project databases (http://www.ncbi.nlm.nih.gov and http://www.hapmap.org, respectively). Two SNPs (rs6590357 and rs3740835) were TagSNPs. The other three SNPs (rs4937391, rs2604204 and rs11221497) were associated with metabolism syndrome [21]. Furthermore, PA patients had a higher prevalence of metabolism syndrome than EH patients. We inferred that PA may share some pathogenic processes with metabolism syndrome, which led us to select the three SNPs for analysis. Genomic DNA was isolated from peripheral blood leukocytes using a PAXgene blood DNA kit (PreAnalytiX™; Qiagen, Germany) and applied as a template for PCR-based genotyping using a previously described *Taq* amplification method in the 7900 HT Fast Real-Time PCR System (Applied Biosystems Inc., USA) [22]. The *Taq*Man SNP genotyping assay primers and probes were chosen based upon specific sequences for the genes of interest available on the Applied Biosystems Inc. website (http://myscience.appliedbiosystems.com).

The five following SNPs in the *KCNJ5* gene were genotyped: rs6590357 (also known as C171T) in the second exon; rs2604204 (6262A/C) in the 3′ untranslated region (3′UTR); rs11221497 (−20559C/G) in the promoter region; and rs3740835 (−10811A/C) and rs4937391 (5126A/G) in the first and second intron region. In addition, three mutations in the second exon (G151R, L168R and T158A) were genotyped. To ensure and verify genotyping quality, the case and control subjects were distributed randomly across the plates. Genotyping was carried out by investigators who were blinded to the phenotypic information, with controls of the known genotype included in each genotyping run. The success rate for genotyping was >99%, and the minor allele frequencies for all subjects was >5%.

### Haplotype Construction

Based on the genotype data for the genetic variations, linkage disequlibrium (LD) analysis was performed along with haplotype-based case-control analysis and haplotype construction. The pairwise LD analysis of the five SNPs was carried out with |D′ values|>0.5 used to assign SNP locations within a single haplotype block. In the haplotype-based case–control analysis, haplotypes with a frequency <0.01 were excluded.

### Statistical Analysis

All statistical analyses were performed with SPSS statistical software, version 16.0 (USA). The data are expressed as mean ± SD or median (interquartile range). Inter-group comparisons were made with the unpaired *t*-test, Mann-Whitney *U* test, or Chi-squared (*χ* ^2^) test. The case-control-based haplotype analysis and Hardy-Weinberg equilibrium test were carried out by the SNPAlyze software, version 2.1 (Dynacom Co. Ltd, Japan). Inter-group differences of SNP genotype or allele frequency were analyzed by the Chi-squared test. Logistic regression analysis was used to calculate odds ratios (ORs) with 95% confidence intervals (CIs). *P*-values <0.05 were considered to indicate statistical significance.

## Results

### Baseline Characteristics of PA and EH Patients

Baseline characteristics of the 235 PA patients and 913 EH patients are summarized in Table 1. There was no significant difference in body mass index (BMI), diastolic blood pressure, serum sodium level, smoking status, or alcohol consumption between the two groups. Compared with the EH group, the PA group had higher serum aldosterone and ARR, but lower serum potassium and PRA overall, in male, and in female subjects. In addition, the PA group had a higher systolic blood pressure than the EH group overall and in male subjects, while the PA group had a lower average age than the EH group in female subjects.

**Table 1:** Baseline characteristics of PA and EH patients.

	Total		Males		Females	
	EH	PA	EH	PA	EH	PA
Number of subjects	913	235	539	146	374	89
Age, years	48±10	47±8^a^	47±10	47±8	50±11	46±8^b^
BMI, kg/m^2^	26.9±3.8	27.1±3.5	27.6±3.5	27.9±3.1	25.9±3.9	25.9±3.9
Current smoker	299	83	295	82	4	1
Alcohol consumption	258	68	253	66	5	2
SBP, mmHg*	140±20	144±19^b^	140±19	146±20^b^	140±21	141±18
DBP, mmHg	93±14	95±13	96±14	97±13	90±14	92±11
Serum potassium, mmol/L	3.9±0.4	3.7±0.4^b^	3.9±0.4	3.7±0.4^b^	3.9±0.4	3.7±0.4^b^
Serum sodium, mmol/L	140.2±2.9	140.4±3.4	140.4±3.0	140.8±3.8	140.1±2.8	139.8±2.6
PRA, ng/mL/h	1.3 (0.5, 2.6)	0.5 (0.2, 0.9)^b^	1.5(0.6, 2.9)	0.6 (0.3, 1.0)^b^	0.9 (0.3, 2.0)	0.4 (0.2, 0.6)^b^
SA, ng/dL	12.3 (8.3, 17.7)	18.6 (14.3, 23.6)^b^	12.4 (8.3, 17.7)	19.0 (15.2, 25.6)^b^	12.1(8.0, 17.6)	17.7 (12.6, 21.9)^b^
ARR, ng/dL/ng/ml/h	9 (5, 22)	37 (23, 81)^b^	8 (4, 16)	32 (20, 78)^b^	13(6, 36)	41 (28, 83)^b^

Data are expressed as mean±SD or median (interquartile range). PA, primary aldosteronism;EH,essential hypertension; PRA, plasma renin activity; SA, serum aldosterone; ARR, aldosterone/renin activity ratio.

* l mmHg = 0.133 kPa.

a *P*<0.05,

b *P*<0.01 for PA group vs. EH group.

### Genotype and Allele Frequencies

Table 2 presents the genotype and allele frequencies of PA and EH patients. In the present study, all five SNPs of the *KCNJ5* gene (rs6590357, rs4937391, rs3740835, rs2604204, and rs11221497) were successfully genotyped and in Hardy–Weinberg equilibrium (*P*>0.05). In contrast, the G151R, L168R, and T158A mutations were not detected in any of the study cases or controls. The EH group and the PA group showed significant differences in the distributions of genotypes and alleles of rs4937391 and rs2604204 overall and in male subjects (all, *P*<0.05), as well as the rs3740835 in male subjects (all, *P*<0.05). The association between the genotype of rs2604204 and male patients with PA remained significant after Bonferroni’s correction (statistical power: 0.623, *P*<0.01). No significant differences were observed between the PA cases and the EH controls for all SNPs tested in the female subjects. In addition, the genotype and allele frequencies between 77 bilateral cases and 39 unilateral cases also were analyzed, but no significant differences were observed (data not shown).

**Table 2:** Genotype and allele frequencies in PA and EH patients.

Variants	genotype/allele	Total		*P*	Males		*P*	Females		*P*
		EH (n/%)	PA (n/%)		EH (n/%)	PA(n/%)		EH (n/%)	PA (n/%)	
rs6590357	CC	707(77.9)	175(75.4)		418(78.1)	108(75.0)		289(77.7)	67(76.1)	
(exon-2)	CT	184(20.3)	55(23.7)	0.344	106(19.8)	35(24.3)	0.296	78(21.0)	20(22.8)	0.928
	TT	16(1.8)	2(0.9)		11(2.1)	1(0.7)		5(1.3)	1(1.1)	
	C	1598(88.1)	405(89.1)	0.634	942(88.0)	251(87.2)	0.683	656(88.2)	154(87.5)	0.805
	T	216(11.9)	59(10.9)		128(12.0)	37(12.8)		88(11.8)	22(12.5)	
rs4937391	AA	411(45.2)	93(39.9)		241(44.9)	47(32.6)		170(45.6)	46(51.7)	
(intron-2)	AG	391(43.0)	98(42.1)	0.038	228(42.4)	68(47.3)	0.011	163(43.7)	30(33.7)	0.198
	GG	108(11.8)	42(18.0)		68(12.7)	29(20.1)		40(10.7)	13(14.6)	
	A	1213(66.6)	284(60.9)	0.021	710(66.1)	162(56.3)	0.002	503(67.4)	122(68.5)	0.775
	G	607(33.4)	182(39.1)		364(33.9)	126(43.7)		243(32.6)	56(31.5)	
rs3740835	AA	42(4.6)	7(3.0)		26(4.8)	1(0.7)		16(4.3)	6(6.7)	
(intron-1)	AC	296(32.4)	75(31.9)	0.522	175(32.5)	38(26.0)	0.014	121(32.3)	37(41.6)	0.116
	CC	575(63.0)	153(65.1)		338(62.7)	107(73.3)		237(63.4)	46(51.7)	
	A	380(20.8)	89(18.9)	0.369	227(21.1)	40(13.7)	0.005	153(20.5)	49(27.5)	0.040
	C	1446(79.2)	381(81.1)		851(78.9)	252(86.3)		595(79.5)	129(72.5)	
rs2604204	AA	422(46.4)	95(40.6)		244(45.4)	50(34.5)		178(47.7)	45(50.6)	
(3'UTR)	AC	385(42.3)	97(41.5)	0.020	228(42.5)	64(44.1)	0.006	157(42.1)	33(37.1)	0.644
	CC	103(11.3)	42(17.9)		65(12.1)	31(21.4)		38(10.2)	11(12.3)	
	A	1229(67.5)	287(61.3)	0.011	716(66.7)	164(56.6)	0.001	513(68.8)	123(69.1)	0.931
	C	591(32.5)	181(38.7)		358(33.3)	126(43.4)		233(31.2)	55(30.9)	
rs11221497	CC	9(1.0)	1(0.4)		5(0.9)	1(0.7)		4(1.1)	0(0)	
(promoter)	CG	191(21.0)	44(18.8)	0.530	116(21.6)	33(22.7)	0.922	75(20.1)	11(12.4)	0.138
	GG	711(78.0)	189(80.8)		417(77.5)	111(76.6)		294(78.8)	78(87.6)	
	C	209(11.5)	46(9.8)	0.314	126(11.7)	35(24.0)	0.866	83(11.1)	11(6.2)	0.050
	G	1613(88.5)	422(90.2)		950(88.3)	255(76.0)		663(88.9)	167(93.8)	

PA, primary aldosteronism;EH,essential hypertension.

To further investigate the effect of the *KCNJ5* gene polymorphisms on PA patients, logistic regression analysis was performed. After adjusting for age, smoking, drinking, and BMI, the CC genotype of rs2604204 was identified as a risk factor of male patients with PA (OR = 2.228, 95% CI: 1.300–3.819, *P* = 0.004).

### Haplotype Analysis

The patterns of pairwise LD between the five common polymorphisms in the *KCNJ5* gene are shown in Table 3. All of the SNPs were considered to be located in a single haplotype block, indicating that all five were suitable for use in a haplotype-based case–control study.

**Table 3:** Patterns of pairwise LD between the five common polymorphisms in the *KCNJ5* gene.

	rs6590357	rs4937391	rs3740835	rs2604204
rs6590357				
rs4937391	0.911			
rs3740835	−0.5089	−0.7955		
rs2604204	0.7909	0.9923	−0.8166	
rs11221497	−0.0886	−0.4432	−0.9046	−0.4212

Values shown represent D′.

Haplotype construction for the five common SNPs yielded a total of 21 haplotypes. Among these 21 haplotypes, the frequencies of six were >1% (Table 4), while the frequencies of the remaining haplotypes were <1%. In EH controls, H1, H2, and H3 were the major haplotypes (37.19, 21.13, and 19.14%, respectively), and H4, H5 and H6 had relatively low frequencies (9.07, 8.08 and 1.32%, respectively). Compared with other haplotypes, only H4 showed a significantly lower frequency in PA patients (*P*<0.05).

**Table 4:** Haplotype analysis in PA cases and EH controls.

	Haplotype					EH	PA	*P*
	rs6590357	rs4937391	rs3740835	rs2604204	rs11221497			
H1	C	A	C	A	G	37.19	36.30	0.717
H2	C	G	C	C	G	21.13	24.41	0.139
H3	C	A	A	A	G	19.14	17.24	0.381
H4	C	A	C	A	C	9.07	6.16	0.034
H5	T	G	C	C	G	8.08	9.74	0.211
H6	T	G	C	C	C	1.32	2.02	0.362

PA, primary aldosteronism;EH,essential hypertension.

### Association between the Genotypes of rs2604204 and Intermediate Phenotypes of Male Patients with PA

There was no significant difference in serum potassium, serum sodium, plasma renin activity, serum aldosterone, or aldosterone/renin activity ratio among the three genotypes of rs2604204 in male subjects with PA (Table 5).

**Table 5:** Association between the genotypes of rs2604204 and intermediate phenotypes of male patients with PA.

Genotype	Serum potassium, mmol/L	Serum sodium,mmol/L	ln(PRA*100), ng/mL/h	lg(SA*10), ng/dL	lg(ARR*10), ng/dL/ng/ml/h
AA (n = 50)	3.7±0.4	141.3±2.1	4.0±0.9	2.3±0.2	2.5±0.4
AC (n = 64)	3.6±0.4	141.0±2.4	3.8±1.0	2.3±0.2	2.6±0.5
CC (n = 31)	3.6±0.4	139.8±6.9	4.0±1.0	2.3±0.2	2.6±0.5
*P* value	0.155	0.197	0.467	0.661	0.321

Data are expressed as mean±SD or median (interquartile range). PRA, plasma renin activity; SA, serum aldosterone; ARR, aldosterone/renin activity ratio.

## Discussion

In this study, the relationship between five common *KCNJ5* polymorphisms (rs6590357, rs4937391, rs3740835, rs2604204 and rs11221497) and sporadic PA were investigated in a patient population from Xinjiang, China. The preliminary results showed that three of the SNPs, rs4937391, rs3740835 and rs2604204, were significantly associated with in male patients with sporadic PA in our study group.

However, only the association between rs2604204 genotype and male patients with PA remained significant after Bonferroni’s correction. Further logistic regression analysis demonstrated that the CC genotype of rs2604204 was a risk factor for the male patients’ sporadic PA, after adjusting for age, sex, and BMI.

Haplotype analysis is a relatively recent approach for elucidating the relationships between candidate genes (*e.g. KCNJ5*) and specific traits (*e.g.* sporadic PA) [23]. In this study, the H4 haplotype of *KCNJ5* was found to be significantly associated with sporadic PA. Furthermore, distributions of the five polymorphisms were found to be in Hardy-Weinberg equilibrium, suggesting that the results of this study are unlikely to be biased by population stratification. Therefore, our results indicate that the rs2604204 SNP may be associated with male patients with sporadic PA in the Chinese.

In 2011, Choi *et al*. [15] identified two somatic *KCNJ5* mutations (*i.e.* G151R and L168R) associated with APA and a germline mutation (*i.e.* T158A) associated with FH-III in an American family. More recently, an additional *KCNJ5* variant, G151E, was identified in European families and associated with FH-III [16]. Subsequently, a larger sample study suggested that the somatic *KCNJ5* mutations (G151R and L168R) may be associated with a more typical PA phenotype [17]. Further studies showed that the frequency of the G151R and L168R mutations were common, which suggested that *KCNJ5* variants might play an important role in the molecular pathogenesis of APA [18]–[19]. In this study, we did not detect the G151R and L168R variants of *KCNJ5* in peripheral blood cells of our study population, either in cases or controls. This finding is consistent with those reported from previous studies [15]–[16]. In addition, we did not detect the T158A mutation in any of the study subjects, but this finding may reflect the fact that FH patients were excluded from our study.

It has been reported that the two somatic variants, G151R and L168R, and the two germline mutations, T158A and G151E, of the *KCNJ5* gene may result in loss of channel selectivity. In addition, the newly reported two somatic variants, delI157 and E145Q, may also result in loss of channel selectivity [24]–[25]. Such an effect is expected to lead to increased sodium conductance and subsequent membrane depolarization, followed by activation of the voltage-gated Ca^2+^ channels and elevated intracellular Ca^2+^ levels. Ultimately, these changes could promote aldosterone secretion and cellular proliferation [15]–[16]. A recent study suggested that the germline G151E mutation produced the most extreme functional effect, facilitating a much larger Na+ conductance than the germline G151R mutation and resulting in rapid onset of Na+-dependent cell lethality [26]. The authors inferred that this increased lethality limited the adrenocortical cell mass, as well as the severity of aldosteronism, *in vivo*. Collectively, however, these findings indicate that different mechanisms exist for different mutations of *KCNJ5* in PA.

The mechanisms by which the rs2604204 SNP might contribute to sporadic PA in the male patients are unknown. The rs2604204 SNP is located in the 3′UTR and plays an important role in the translation, transcription, localization, and stability of mRNA. Several studies have shown that 3′UTRs mediate gene regulation mainly at the translation level, and mRNA stability by providing docking sites for microRNAs and RNA binding proteins [27]–[29]. Recently, Gomez-Benito *et al*. demonstrated that 3′UTRs can also influence gene transcription [30]. Changes in 3′UTR length, sequence, or location have been shown to influence gene expression, cell proliferation, and survival under physiological conditions, and lead to cancer development in pathologic conditions [31]. Further study is required to clarify the precise role of the rs2604204 SNP of *KCNJ5* in the pathogenesis of PA in male patients.

Boulkroun *et al.* reported that KCNJ5 mutation carriers are more likely to be females [17], and another study also showed that APAs without KCNJ5 mutations had a higher prevalence in males than females [25]. However, in our study, rs2604204 variant of *KCNJ5* was associated with male PA patients. The inconsistent results may be attributed to the difference in the rate at which these mutations occur in female or male patients with PA. Of course, this result requires further validation with a larger sample size.

In summary, we found that a common SNP of *KCNJ5* (rs2604204) was associated with sporadic PA in Chinese males. This finding suggests that common genetic variations of *KCNJ5* may be involved in the pathogenesis of sporadic PA in Chinese males. However, further functional analyses of the rs2604204 mutation are necessary to clarify the functional defects caused by the genetic findings.
